# Functional roles of cadherin, aminopeptidase-N and alkaline phosphatase from *Helicoverpa armigera* (Hübner) in the action mechanism of *Bacillus thuringiensis* Cry2Aa

**DOI:** 10.1038/srep46555

**Published:** 2017-05-10

**Authors:** Man Zhao, Xiangdong Yuan, Jizhen Wei, Wanna Zhang, Bingjie Wang, Myint Myint Khaing, Gemei Liang

**Affiliations:** 1 State Key Laboratory for Biology of Plant Diseases and Insect Pests, Institute of Plant Protection, Chinese Academy of Agricultural Sciences, Beijing 100193, China

## Abstract

A pyramid strategy combining the Cry1A and Cry2A toxins in Bt crops has been widely used throughout the world to delay pest adaption to transgenic crops and broaden the insecticidal spectrum. Midgut membrane-bound cadherin (CAD), aminopeptidase-N (APN) and alkaline phosphatase (ALP) are important for Cry1A toxicity in some lepidopteran larvae, but the proteins that bind Cry2A in the midgut of target insects and their role in the Cry2A mechanism of action are still unclear. In this study, we found that heterologously expressed CAD, APN4 and ALP2 peptides from the midgut of *Helicoverpa armigera* could bind to the Cry2Aa toxin with a high affinity. Additionally, the efficiency of Cry2Aa insecticidal activity against *H. armigera* larvae was obviously reduced after the genes encoding these proteins were silenced with specific siRNAs: CAD- and ALP2-silenced larvae showed significantly similar reductions in mortality due to the Cry2Aa toxin (41.67% and 43.06%, respectively), whereas a larger reduction in mortality was observed in APN4-silenced larvae (61.11%) than in controls. These results suggest that CAD, APN4 and ALP2 are involved in the mechanism of action of Cry2Aa in *H. armigera* and may play important functional roles in the toxicity of the Cry2Aa toxin.

Selected *cry* genes encoding insecticidal crystal proteins from the soil bacterium *Bacillus thuringiensis* (Bt) have been successfully transferred to genetically modified (GM) crops to produce toxic proteins because these proteins show specific toxicity to certain insect pests, such as Lepidoptera, Diptera, and Coleoptera, but do not harm non-target organisms^1^,^2^,^3^,^4^,^5^. In addition to their economic benefits, GM crops also have clearly delivered substantial agricultural and environmental benefits to farmers and society. Therefore, the global cultivated acreage of GM crops increased rapidly following their first commercialization in 1996, covering 1.7 million hectares, to 179.7 million hectares in 2015, and Bt crops, such as Bt cotton and Bt maize, lead the world in the adoption of GM crops, covering more than 78 million hectares^6^,^7^,^8^,^9^.

However, similar to the history of synthetic pesticides, long-term application of Bt crops and Bt spray products exerts high selection pressure on their target insects, resulting in some insects evolving resistance to Bt insecticidal proteins. As a result, the durability and effectiveness of Bt crops have diminished^10^,^11^,^12^. Thus, a pyramid strategy in which at least two sufficiently different Bt toxins are expressed in a single plant was proposed for use in resistance management programs. Two-toxin crops are generally expected to improve the efficacy of Bt, broaden the insecticidal spectrum, delay the evolution of target pest resistance and increase the duration of benefits from transgenic crops^13^. For example, two-toxin Bt cotton, in which both the Cry1A and Cry2A toxins are produced, has been widely used in many countries to replace first-generation Bt cotton, producing only the Cry1A toxin. However, only first-generation Bt cotton has been allowed for commercial production in China since 1996^14^,^15^.

The cotton bollworm, *Helicoverpa armigera* (Hübner; Lepidoptera:Noctuidae), is a major polyphagous pest of cotton and other crops in China, and the introduction of Bt cotton expressing Cry1Ac has effectively suppressed regional outbreaks of *H. armigera*, not only on cotton but also on other host crops^16^,^17^. However, the discovery of resistant *H. armigera* populations under laboratory and field conditions may signal an early warning for more serious problems, such as control failure of Bt cotton^18^,^19^,^20^,^21^. Cry1Ac and Cry2Ab are considered to represent a good combination of Bt toxins because they show low amino acid homology and might ideally target different binding sites in the larval midgut^22^,^23^. In contrast with Cry1A proteins, Cry2A toxins can also effectively control lepidopteran pests other than *H. armigera*, such as *Spodoptera exigua* and *Agrotis ypsilon*^24^. Consequently, introduction of Cry1A/Cry2A Bt cotton in China is expected to provide more effective control activity against many target lepidopteran pests and assist in delaying the development of insect resistance to Bt toxic proteins^25^,^26^.

After being ingested by susceptible lepidopteran larvae, the Cry protoxin is solubilized and activated in the midgut. It then goes through a complex series of interactions with midgut membrane proteins, finally leading to cell death and killing the larvae^27^,^28^. Several membrane proteins have been characterized as functional receptors for the Cry1A toxin, including cadherin (CAD), aminopeptidase-N (APN), alkaline phosphatase (ALP) and ATP-binding cassette (ABC) transporter protein^29^,^30^,^31^, whereas the binding receptors of the Cry2A toxin and their potential roles in the mode of action of the Cry2A protein remain incompletely characterized. To determine the roles of CAD, APN and ALP from the midgut of *H. armigera* in the Cry2Aa toxin mechanism of action, we studied the binding characteristics of the Cry2Aa toxin with recombinant *H. armigera* CAD, APN4 (a cluster of lepidopteran APNs) and ALP2 (a cluster of lepidopteran ALPs) peptides^32^,^33^. Then, we investigated their roles in Cry2Aa toxicity by suppressing their transcription with siRNAs. Based on our results, we hypothesize that CAD, APN4 and ALP2 may function as receptors for the Cry2Aa toxin in *H. armigera* and play important functional roles in the mechanism of action of the Cry2Aa toxin.

## Results

### Toxicity of Cry1Ac and Cry2Aa to the susceptible *H. armigera* strain

The results of insect assays showed that the Cry1Ac and Cry2Aa toxins exhibited similar toxicity against first-instar *H. armigera* larvae. The LC_50_ values of these two toxins were 1.20 μg/cm^2^ (95% fiducial limits, 0.79–1.92) and 1.05 μg/cm^2^(95% fiducial limits, 0.59–1.91), respectively.

### Expression and purification of HaCAD, HaAPN4 and HaALP2 and determination of polyclonal antibody specificity

**Figure 1:**
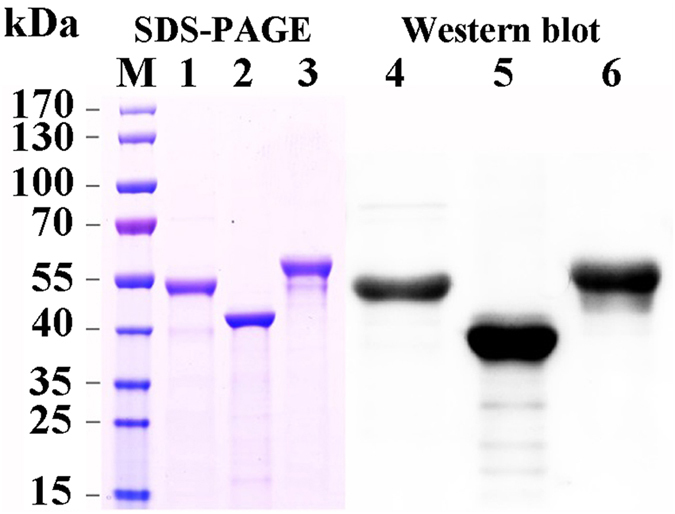
Quality of the purified recombinant CAD, APN4 and ALP2 proteins and Western blot analysis of these recombinant proteins using their respective polyclonal antibodies. Lane M: protein marker; lanes 1–3: Purified recombinant CAD, APN4 and ALP2 proteins; lanes 4–6: The purified CAD, APN4 and ALP2 fragments were detected with the corresponding polyclonal antibodies.

To further characterize the binding of CAD, APN4 and ALP2 from *H. armigera* to the Cry2Aa toxin, three fragments of these proteins were expressed in *E. coli* BL21 (DE3) cells and purified from the total crude proteins. The expressed peptides were evaluated through SDS-PAGE, and the results demonstrated that the expressed CAD, APN4 and ALP2 proteins exhibited the expected sizes of 47 kDa, 40 kDa and 51 kDa, respectively, and that there was only one main visible band for these three proteins after purification (Fig. 1, lane 1, lane 2 and lane 3). The purified proteins were detected with the corresponding antibodies against HaCAD, HaAPN4 and HaALP2, and the results indicated that all three purified recombinant proteins could strongly hybridize with the corresponding polyclonal antibodies (Fig. 1, lane 4, lane 5 and lane 6).

### Western and ligand blotting detection

**Figure 2:**
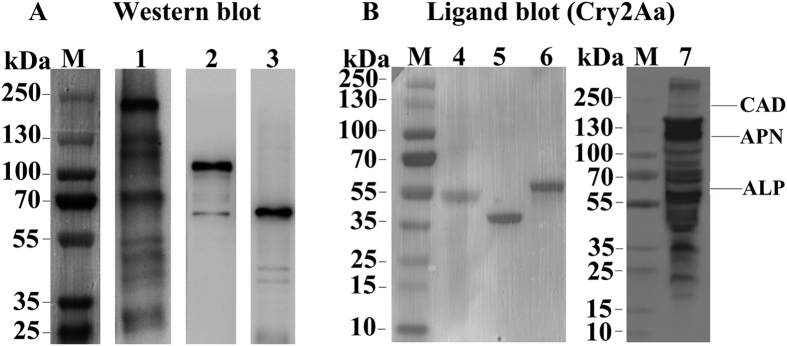
Western blot analysis of anti-CAD, anti-APN4 and anti-ALP2 antibodies using BBMV proteins from *H. armigera* larvae (**A**) and ligand blot analysis of Cry2Aa with the recombinant CAD, APN4 and ALP2 proteins or BBMV proteins (**B**). Lane M: protein marker; lanes 1–3: CAD, APN4 and ALP2 antibody binding of BBMV from fifth-instar *H. armigera* larvae; lanes 4–6: Cry2Aa toxin binding by the recombinant CAD, APN4 and ALP2 proteins; lane 7: Cry2Aa binding with BBMV proteins.

The CAD, APN4 and ALP2 proteins in brush border membrane vesicle (BBMV) samples obtained from the midguts of *H. armigera* were detected by western blotting using anti-HaCAD, anti-HaAPN4 and anti-HaALP2, and the results revealed an apparent size for the CAD protein of 210 kDa, for the APN4 protein of 120 kDa and for the ALP2 protein of 65 kDa in the BBMV samples (Fig. 2A, lane 1, lane 2 and lane 3). To determine the binding of the Cry2Aa toxin to the CAD, APN4 and ALP2 proteins from the midguts of *H. armigera*, ligand blotting analysis of the biotinylated Cry2Aa toxin was performed using BBMV protein or heterologously expressed recombinant HaCAD, HaAPN4 and HaALP2 proteins. The results showed that ligand blot could detect several binding bands, included binding proteins of 210, 120 and 65 kDa for Cry2Aa toxin in the BBMV proteins from susceptible cotton bollworm (Fig. 2B, lane 7), and all the three purified recombinant proteins could also bind to the Cry2Aa toxin (Fig. 2B, lane 4, lane 5 and lane 6).

### ELISA binding assays of Cry2Aa with HaCAD, HaAPN4 and HaALP2

**Figure 3:**
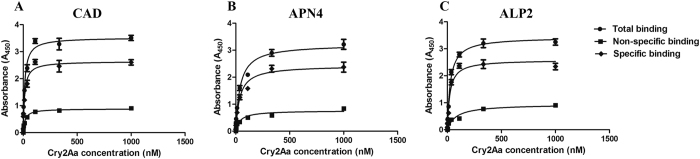
ELISAs to assess the binding the Cry2Aa toxin with the recombinant CAD, APN4 and ALP2 fragments. Binding ELISAs were performed by fixing 1 μg of the CAD (**A**), APN4 (**B**) and ALP2 (**C**) fragments per well in ELISA plates, followed by incubation with different concentrations of the biotinylated Cry2Aa toxin. *K*_*d*_ values obtained through SigmaPlot 12.5 analysis are indicated within the graphs.

To further determine the specific binding characteristics of the Cry2Aa toxin in relation to the recombinantly expressed HaCAD, HaAPN4 and HaALP2 proteins, we tested their binding affinities through ELISA binding assays. The results showed that the Cry2Aa toxin bound to the recombinant HaCAD, HaAPN4 and HaALP2 proteins with high affinities of 12.28 nM, 36.82 nM and 22.91 nM, respectively (Fig. 3A–C).

### Effects of CAD, APN4 and ALP2 knockdown on their transcript levels and Cry2Aa-induced larval mortality in *H. armigera*

**Figure 4:**
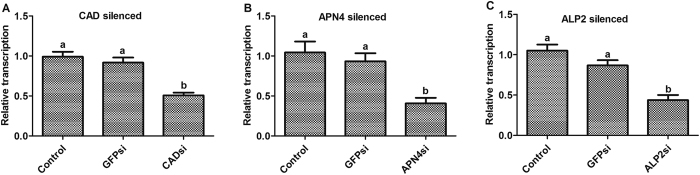
Transcript levels of CAD, APN4 and ALP2 after feeding specific siRNA constructs to early second-instar larvae of *H. armigera*. Transcript abundance was determined via qPCR. Bars represent the means and standard errors for 3 individual samples (**A**–**C**).

**Figure 5:**
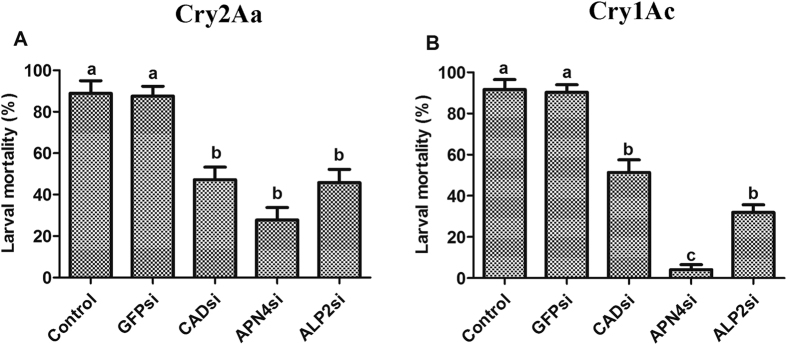
Effects of CAD, APN4 and ALP2 gene silencing on the toxicities of the Cry1Ac and Cry2Aa toxins. CAD-silenced, APN4-silenced, ALP2-silenced and control larvae were exposed to diets containing 8 μg/cm^2^ of the Cry2Aa (**A**) or Cry1Ac toxin (**B**), and mortality was recorded after 7 days. Bars represent the means and standard errors determined from 3 replicates. Different letters indicate a significant difference at a P value < 0.05.

RNAi efficiency was investigated via qPCR, and the results showed that the transcription of *HaCAD, HaAPN4* and *HaALP2* in larvae fed diets containing *HaCAD-, HaAPN4-*or *HaALP2*-siRNA was significantly reduced by 48.33% (*P* = 0.002), 63.67% (*P* = 0.013) and 61.33% (*P* = 0.002), respectively, compared with that in larvae fed control diets with only RNase-free H_2_O or *GFP*-siRNA (Fig. 4A–C). The CAD- and ALP2-silenced larvae showed significantly similar reductions of the Cry2Aa toxin-induced mortality rate (41.67% (*P* = 0.004) and 43.06% (*P* < 0.001), respectively), whereas a greater reduction in mortality was observed in the APN4-silenced larvae (61.11%, *P* = 0.003) compared with the controls (Fig. 5A). The Cry1Ac toxin was used as a positive control, and the results showed that the most significant reduction of mortality was observed in APN4-silenced *H. armigera* larvae, which exhibited an 87.50% reduction (*P* < 0.001), followed by ALP2-silenced larvae (59.72% mortality reduction, *P* < 0.001) and CAD-silenced larvae (40.28% reduction, *P* < 0.001; Fig. 5B).

## Discussion

In the present study, the Cry2Aa and Cry1Ac toxins showed similar toxicity to the susceptible strain of *H. armigera* (for Cry2Aa, LC_50_ = 1.05 μg/cm^2^; for Cry1Ac, LC_50_ = 1.20 μg/cm^2^). In addition to *H. armigera*, Cry2A toxins (Cry2Aa and Cry2Ab) also have been shown to exhibit high efficiency against several other lepidopteran pests^34^,^35^,^36^,^37^. Therefore, the introduction of Cry1A/Cry2A Bt cotton in China is expected to be more effective against target pests and to broaden the insecticidal spectrum compared with first-generation Bt cotton.

A key step in the mechanism of action of Cry toxins in lepidopteran pests is the interaction of activated Cry proteins with specific binding sites in the BBMVs of the larval midgut, and reduced binding of Cry toxins to these membrane binding receptors is generally related to resistance of target pests to Bt toxins^11^,^26^,^28^,^38^,^39^,^40^. The insect midgut membrane protein CAD and the highly abundant glycosyl phosphatidylinositol (GPI)-anchored APN and ALP proteins have been characterized as functional binding receptors for Cry1A toxins in several lepidopteran insects^28^,^41^,^42^,^43^. However, limited studies have focused on defining the role of the insect membrane proteins CAD, APN and ALP in relation to Cry2A toxins to date^44^,^45^.

In lepidopteran pests, insect midgut CAD, APN and ALP proteins are important binding receptors for several classes of Cry1A toxins^2^,^26^,^43^,^46^,^47^,^48^. GPI-anchored APN and ALP proteins first bind to protease-activated Cry1A toxins with a low affinity (101 nM for APN, 267 nM for ALP) and subsequently carry the toxins to the microvilli membrane of the larval midgut, where the toxins bind to CAD with a high affinity (1 nM). Then, CAD promotes oligomerization of Cry toxins, and APN and ALP interact with the oligomeric Cry structure with a high affinity (0.6 nM for APN, 0.5 nM for ALP), which causes membrane insertion and pore formation, ultimately leading to osmotic lysis of midgut cells^28^,^41^,^42^. The results of ligand blot and western blot assays conducted in this study indicated that the 210, 120 and 65 KDa proteins from *H. armigera* larval midgut BBMV could recognize by Cry2Aa toxin and the antibodies that against CAD, APN and ALP, respectively. In addition, the Cry2Aa toxin could bind to all the three expressed recombinant proteins (HaCAD, HaAPN4 and HaALP2; Fig. 2B). The binding affinities of HaCAD and HaALP2 with Cry2Aa were 3.00-fold and 1.61-fold higher, respectively, than that of HaAPN4 (Fig. 3). However, the binding affinity results indicated much lower affinities than reported previously^28^,^49^,^50^. This difference may be due to the different toxin proteins examined or to the fact that the CAD, APN and ALP proteins used in this study were heterologously expressed as recombinant proteins. Previous reports of findings in *H. armigera* and *H.virescens* revealed that the binding of Cry proteins to APN and ALP relies on an *N*-acetylgalactosamine interaction^51^,^52^. However, in the present study, the expressed recombinant HaAPN4 and HaALP2 proteins from *E. coli* were not glycosylated, which explains the low binding affinity of the Cry2Aa toxin for the expressed recombinant APN and ALP proteins.

Cry1A and Cry2A are the two most widely used Cry toxins in pyramided transgenic cotton based on reports indicating that Cry1A and Cry2A exhibit different binding receptor proteins and different modes of action^22^,^23^,^53^,^54^. However, further research indicated that Domain II of Cry2A shows similar binding sites to Cry1A^23^,^55^, and some cross-resistance between these two toxin types has been documented. This finding implies that they may share a common receptor, although cross-resistance can be independent of toxins sharing receptors^19^,^44^,^56^,^57^,^58^. In the current study, after silencing the *HaCAD, HaAPN4* and *HaALP2* genes using specific siRNAs *in vivo*, CAD, APN4 and ALP2 transcript levels were found to be suppressed (Fig. 4). Compared with a positive control using the Cry1Ac toxin, a similar increase in the tolerance of *H. armigera* to Cry2Aa was observed (Fig. 5). Therefore, these three membrane-bound midgut proteins may play similar significant roles in the mechanism of action of the Cry2Aa toxin in *H. armigera*.

Recently, CAD was confirmed to be involved in the action of the Cry2Aa toxin in *S. exigua*, serving as a functional receptor for both the Cry2Aa and Cry1Ac toxins. These two toxins did not compete for the same binding sites, which suggests that they may bind to diverse CAD protein epitopes in this species^45^,^59^. It has also been shown that ALP2 plays a dominant role in the susceptibility of *S. exigua* to the Cry2Aa toxin^60^. Among the several clusters of APNs present in lepidopteran insects, APN1 has been reported as an important functional receptor for Cry1Ac in several lepidopteran species^42^,^44^,^61^, and APN3 and APN6 serve as functional candidate receptors for Cry1Ca in *S. exigua*^62^. However, there is no direct or conflict evidence of the roles of other APNs in the mechanisms of Cry1A, Cry2A or other Cry toxins^46^. In the present study, we also found that CAD and ALP played significant roles in the action of the Cry2Aa toxin, similar to those associated with the Cry1Ac toxin. Silencing of APN4 in *H. armigera* had less of an effect on the toxicity of Cry2Aa (61.11% reduction in larval mortality) than on the Cry1Ac toxin (87.50%), which might indicate that APN exerts a greater effect on the toxicity of Cry1Ac than Cry2Aa (Fig. 5). Our results suggested that CAD, APN4 and ALP2 are involved in the action of the Cry2Aa toxin in *H. armigera* and play important functional roles in the toxicity of Cry2Aa. Therefore, we hypothesize that CAD, APN4 and ALP2 may function as receptors for Cry2Aa in *H. armigera*. However, to determine whether the Cry1Ac and Cry2Aa toxins exhibit the same binding sites on CAD, APN or ALP, more compete binding experiments are needed.

## Materials and Methods

### Insect rearing

The susceptible *H. armigera* 96S strain was initiated from 20 pairs of adults collected in Xinxiang County, Henan Province, China, in 1996. After collection, the moths were maintained in the laboratory at 27 ± 1 °C, under 70 ± 10% relative humidity (RH) and a photoperiod of 14: 10 light:dark (L:D) h. The larvae of the laboratory colony were reared on an artificial diet, and adults were provided with a 10% sugar solution^63^. Both larvae and adults were maintained in the laboratory without exposure to any insecticides.

### Bt toxins and insect bioassays

The Bt toxins Cry1Ac and Cry2Aa used in this study were purchased from Envirotest-China (an agent of Envirologix Inc., Portland, ME, USA; www.envirotest-china.com); the details of their production and purification were described by Li *et al*.^64^. The susceptibility of 96S larvae to Bt proteins was evaluated through diet overlay bioassays^65^. The Cry1Ac or Cry2Aa protein was dissolved and diluted with 50 mM Na_2_CO_3_ (pH 10.5) to obtain various concentrations of either protein, after which 75 μL of solutions corresponding to each dose was overlaid on the diet surface in each well of 24-well plates (TianJin Xiangyushun Co., TianJin, China) and allowed to air-dry. For controls, the same volume of 50 mM Na_2_CO_3_ (pH 10.5) was poured onto the surface of the diet. A total of 72 neonates of the 96S strain were then individually placed on the dried diet surface in 24-well plates for each treatment in three replicates. When assessed 7 days later, larvae that were dead or did not grow to the third instar were considered to be dead. Larval mortality was recorded to calculate the LC_50_.

### Midgut preparation, total RNA extraction and cDNA synthesis

The midguts of 20 fifth-instar *H. armigera* larvae were dissected and washed with an ice-cold 0.7% NaCl solution, then immediately frozen in liquid nitrogen and stored at −80 °C for RNA extraction. Total RNA was extracted from the midguts using the TRIzol reagent (Invitrogen, CA, USA) according to the manufacturer’s instructions. The purity of the total RNA was evaluated by measuring the 260/280 and 260/230 ratios with a NanoVue spectrophotometer (GE Healthcare, CT, USA), and RNA integrity was evaluated through 1% agarose gel electrophoresis. Then, genomic DNA was removed the RNA using DNase I (TakaRa, Dalian, China), and first-strand cDNA was immediately synthesized from 4 μg of total RNA using the SuperScript^TM^ IIIFirst-Strand Synthesis Kit (Invitrogen, CA, USA).

### Expression and purification of recombinant proteins and polyclonal antibody production

According to the nucleotide sequences of the *cad* gene (GenBank accession no. AF519180), the *apn4* gene (GenBank accession no. AY181026) and the *alp2* gene (GenBank accession no. EU729323) of *H. armigera*, three pairs of primers containing *BamH*I and *Hind*III restriction sites at the 5′ends (Table S1) were designed to amplify partial fragments of CAD, APN4, and ALP2 corresponding to bases 3649–4383, 1084–1980 and 532–1377, respectively, which encode amino acid residues 1217–1461, 361–660, and 177–459. The conditions for PCR amplification included a pre-denaturing step at 94 °C for 3 min, followed by 35 cycles of denaturation at 94 °C for 1 min, annealing at 55 °C for 30 s and extension at 72 °C for 1.5 min, with a final extension at 72 °C for 10 min. Subsequently, the PCR products were gel purified with the AxyPrep DNA gel extraction kit (Axygen Scientific, CA, USA) and subcloned into the pEASY-T3 vector (TransGen, Beijing, China), then transformed into *Escherichia coli* Trans-T1 cells (TransGen, Beijing, China). Next, the recombinant T3 vectors were digested with *BamH*I and *Hind*III (TaKaRa Bio Inc., Dalian, China) at 37 °C for 2 h, and the gene fragments were then subcloned into the His-tag expression vector pET-30a (+) (Addgene, MA, USA), which had previously been digested with the same restriction enzymes to generate the pET-CAD, pET-APN4 and pET-ALP2 recombinant plasmids. The coding sequences and clone orientations were confirmed by sequencing.

The recombinant plasmids described above were transformed into *E.coli* BL21 (DE3) (Tiangen, Beijing, China) to express the recombinant HaCAD, HaAPN4 and HaALP2 proteins. Positive transformed clones were selected and cultured at 37 °C in LB medium supplemented with 100 mg/ml ampicillin. The three recombinant proteins were induced by adding isopropyl β-D-1-thiogalactopyranoside (IPTG) to a final concentration of 1 mM. Then, the cultured cells were harvested via centrifugation at 7,000 rpm at 4 °C for 30 min, re-suspended and lysed in phosphate-buffered saline (NaCl 137 mM, KCl 2.7 mM, Na_2_HPO_4_ 4.3 mM, KH_2_PO_4_ 1.4 mM, pH 7.4). After sonication on ice for 15 min, total crude protein was isolated from the lysate through centrifugation at 18,000 rpm at 4 °C for 30 min. For protein purification, the expressed recombinant proteins were purified with a nickel-nitrilotriacetic acid (Ni-NTA) affinity column (Genscript Biology Co., NJ, USA) and eluted from the column with elution buffer (8 M urea, 100 mM NaH_2_PO_4_, 10 mM Tris-HCl, 500 mM imidazole, pH 8.0), then refolded through a gradient of decreasing concentrations of urea (8 M, 6 M, 4 M, 2 M, 1 M) and desalted with a dialysis bag (molecular weight cutoff 10,000–14,000; Sigma, MO, USA) against 10 mM PBS (pH 7.4) overnight at 4 °C. The purity of these three recombinant proteins was verified in 4–20% gradient SDS-PAGE gels (Genscript Biology Co., NJ, USA), and total protein concentrations were estimated via the Bradford method using bovine serum albumin (BSA, Sigma, MO, USA) as the standard protein^66^. The purified HaCAD, HaAPN4 and HaALP2 proteins were used as antigens in rabbits, and the sera from immunized rabbits were collected and purified to generate polyclonal antibodies.

### Western and ligand blotting assays

The midguts of fifth-instar *H. armigera* larvae were prepared as described previously to isolate brush border membrane vesicles (BBMVs) through the differential magnesium precipitation method^67^, and the protein concentration was measured. To detect the CAD, APN4 and ALP2 proteins, total BBMV proteins (20 μg) or purified recombinant proteins (10 μg) were dissolved in loading buffer (LaBest, Beijing, China), then heat denatured and separated in 4–20% gradient SDS-PAGE gels (Genscript Biology Co.,China). Next, the proteins were transferred to polyvinylidene fluoride (PVDF) filters (Millipore, MA, USA) in transfer buffer (39 mM Glycine, 48 mM Tris, 0.037% SDS, and 20% methanol), and the filters were subsequently blocked overnight in PBS (135 mM NaCl, 2 mM KCl, 10 mM Na_2_HPO_4_, and 1.7 mM KH_2_PO_4_, pH 7.4) containing 0.05% Tween-20 and 5% skim milk with constant agitation at 120 rpm. After being washed three times (10 min each time) with PBST (PBS containing 0.05% Tween-20), the filters were probed for 1 h at room temperature with polyclonal antibodies (1:4,000 dilution) against HaCAD, HaAPN4 and HaALP2 (separately), followed by another three washes. Then, the filters were probed with horseradish peroxidase (HRP)-conjugated anti-rabbit IgG (ZSGB-BIO, Beijing, China) at a 1:10,000 dilution for 1 h at room temperature. After another series of washes, the filters were developed using the EasySee Western Blot Kit (TransGen, Beijing, China) and photographed with an ImageQuant LAS4000mini system (GE Healthcare, Japan).

Prior to ligand blotting assays, the purified Cry2Aa toxin was labeled using a Biotin Labeling Kit (Elabscience, Wuhan, China) according to the manufacturer’s instructions. Then, the BBMV proteins from *H. armigera* larval midgut or purified HaCAD, HaAPN4 and HaALP2 proteins were separated, transferred and blocked following the same methods described above for western blotting. After being washed three times with PBST, the filters were incubated for 1 h in blocking buffer with biotinylated Cry2Aa toxin (5 μg/mL), then washed again and then probed with horseradish peroxidase (HRP)-conjugated streptavidin (Thermo Fisher Scientific, MA, USA) in blocking buffer (1: 10,000 dilution) for 1 h at room temperature. The subsequent procedures for detection were as same as those described for western blotting.

### Binding ELISAs

The binding of the Cry2Aa toxin to the recombinant purified HaCAD, HaAPN4 and HaALP2 proteins was tested via enzyme-linked immunosorbent assays (ELISA). Individual wells of 96-well ELISA plates (Costar 9018, Sigma, USA) were coated overnight at 4 °C with 1 μg of the purified HaCAD, HaAPN4 or HaALP2 protein in a final volume of 100 μl of PBS for each well. On the following day, the plates were washed three times (10 min each) with 200 μl of PBST in each well to remove unbound fragments and then blocked with 200 μl of blocking buffer (PBST containing 1% BSA) for 2 h at room temperature, with shaking at 80 rpm. Next, each well was washed three times with 200 μl of PBST, followed by the addition of different concentrations of the biotinylated Cry2Aa toxin to each well in a final volume of 100 μl of blocking buffer. The plates were subsequently incubated at room temperature for 1 h with constant shaking at 80 rpm. After incubation, unbound toxins in each well were removed through washing steps, and the wells were then incubated with HRP-conjugated streptavidin (Thermo Fisher Scientific, MA, USA) in blocking buffer (1:10,000 dilution) for 1 h at room temperature. The plates were subsequently washed again, and 100 μl of freshly prepared 3,3′,5,5′-tetramethylbenzidine (TMB) ELISA substrate was added to each well. After incubation for 30 min at room temperature, the reaction was terminated by adding 50 μl of 0.5 M H_2_SO_4_ and the optical density (OD) values were read with a multi-mode microplate reader (FlexStation 3, Molecular Devices, USA). Nonspecific binding was evaluated in the presence of excess unlabeled Cry2Aa toxin, and specific binding was calculated by subtracting nonspecific binding from total binding. The binding data were analyzed using SigmaPlot v.12.5software (Systat Software, USA).

### Silencing of CAD, APN and ALP in *H. armigera* through RNA interference

The roles of HaCAD, HaAPN4 and HaALP2 in the susceptibility of *H. armigera* to Cry2Aa were tested with an RNA interference (RNAi) technique using sequence-specific small interfering RNAs (siRNA) synthesized by Invitrogen (USA). The siRNA sequences are listed in Table S1. Lyophilized siRNAs targeting *HaCAD, HaAPN4, HaALP2* and *GFP* (negative control) were dissolved with RNase-free H_2_O to a final concentration of 0.015 nM. Then, 75 μl of the suspensions or RNase-free H_2_O (positive control) was poured into the wells of 24-well plates into which 1 ml of the solidified artificial diet had previously been added and allowed to air-dry. Subsequently, early second-instar 96S larvae that had been starved for 12 h were separately transferred into each well of the 24-well plates and allowed to feed for 2 days. The larvae from the control and siRNA-fed treatments were then randomly divided into two groups. One group was collected to assess the transcript levels of *HaCAD, HaAPN4* and *HaALP2* using quantitative real-time PCR (qPCR) as described below, and the other group was individually transferred to a newly prepared artificial diet containing Cry1Ac (8 μg/cm^2^) or Cry2Aa (8 μg/cm^2^) to evaluate the effect of knockdown of *HaCAD, HaAPN4* and *HaALP2* on the toxicity of the Cry1Ac and Cry2Aa toxins. The larval mortality of the latter group was recorded 7 days later. Each treatment included three replicates, and each replicate included 24 *H. armigera* larvae.

### qPCR analysis of the suppression of *HaCAD, HaAPN4* and *HaALP2*

The suppression of *HaCAD, HaAPN4* and *HaALP2* transcript levels was analyzed via qPCR to evaluate the effectiveness of the silencing method. Midgut isolation, total RNA extraction and cDNA synthesis were performed as described previously. Each treatment included three biological samples, and each sample included 20 pooled midguts of *H. armigera* larvae. qPCR was conducted using the cDNA template from each biological sample, and each sample was measured in triplicate in anABI 7500 fast RT-PCR system (Applied Biosystems, Foster City, CA, USA) with the SuperReal PreMix Probe RT-PCR Kit (Tiangen, Beijing, China). qPCR amplifications were carried out in a final volume of 20 μl, containing 1 μl of cDNA (200 ng), 0.6 μl of each primer (10 μM) listed in Table S1, 10 μl of 2 × SuperReal PreMix (probe), 0.4 μl of Probe, 0.4 μl of 50 × RO × Reference Dye and 7.0 μl of RNase-Free ddH_2_O. The conditions for qPCR involved an initial denaturation at 95 °C for 15 min, followed by 40 cycles of 95 °C for 3 s and 60 °C for 30 s. The relative transcript levels of the target genes were first normalized to the reference genes β-actin (Accession no. EU527017.1) and glyceraldehydes-3-phosphate dehydrogenase (GAPDH; Accession no. JF417983.1), and then calculated using the comparative Ct (2^−ΔΔCt^) method^68^.

### Data analysis

The concentration of the Cry1Ac or Cry2Aa toxin causing 50% mortality (LC_50_) and the 95% fiducial limits were calculated via Probit analysis (Polo-PC; LeOra Software, Petaluma, CA, USA). Comparisons to determine significance in the RNAi assays, including assessment of the relative transcription of *HaCAD, HaAPN4* and *HaALP2* and larval mortality on diets treated with or without Bt toxins, were performed (the larval mortality was arcsine transformed before comparison) via one-way analysis of variance (ANOVA) with Tukey’s test for multiple comparisons using SPSS v.18.0 (SPSS Inc., Chicago, IL, USA).

## Additional Information

**How to cite this article**: Zhao, M. *et al*. Functional roles of cadherin, aminopeptidase-N and alkaline phosphatase from *Helicoverpa armigera* (Hübner) in the action mechanism of *Bacillus thuringiensis* Cry2Aa. *Sci. Rep.* **7**, 46555; doi: 10.1038/srep46555 (2017).

**Publisher's note:** Springer Nature remains neutral with regard to jurisdictional claims in published maps and institutional affiliations.

## Supplementary Material

Supplementary Information
