# Supplementary material for: Functional roles of cadherin, aminopeptidase-N and alkaline phosphatase from Helicoverpa armigera (Hübner) in the action mechanism of Bacillus thuringiensis Cry2Aa
Source: Sci Rep. 2017 May 10;7:46555. doi: 10.1038/srep46555 (PMC5424343; doi:10.1038/srep46555)
Supplement: Supplementary Information [file srep46555-s1.pdf]

Supplementary information

Functional roles of cadherin, aminopeptidase-N and alkaline phosphatase from *Helicoverpa armigera* (Hübner) in the action mechanism of *Bacillus thuringiensis* Cry2Aa

Man Zhao, Xiangdong Yuan, Jizhen Wei, Wanna Zhang, Bingjie Wang, Myint Myint Khaing, Gemei Liang*

State Key Laboratory for Biology of Plant Diseases and Insect Pests, Institute of Plant Protection, Chinese Academy of Agricultural Sciences, Beijing 100193, China

*Corresponding Author: Gemei Liang, State Key Laboratory for Biology of Plant Diseases and Insect Pests, Institute of Plant Protection, Chinese Academy of Agricultural Sciences, Beijing, China. Tel.: +86 0106281 5929; e-mail: gmliang@ippcaas.cn

Table S1. Primers used for protein expression, qPCR and siRNA sequences used for RNAi

Purpose	Description	Sequences(5'-3')
Protein expression	HaCAD-F	CGGGATCCACGATTCGTGCTACGGACGGT
	HaCAD-R	CCGCTCGAGTCAGGTACACCTTCACTTCCG
	HaAPN4-F	CGGGATCCAACCTCGTCACCTGCTTCTGG
	HaAPN4-R	CCGCTCGAGAGTAATCGGTCTCATCCCTCA
	HaALP2-F	CGGGATCCACGACGACTCGTATCACTCAC
	HaALP2-R	CCGCTCGAGCGTCCACGTGCGACTCCCAGT
qPCR	HaCAD-F	GCTACCAGCGCCAGTCCTT
	HaCAD-R	GGCCCTTAGCTGTATATTCTGGAA
	Probe	CACGCAGAGCCATCACATGCTGG
	HaAPN4-F	TCGACAGCTGGGTCCAGAAC
	HaAPN4-R	GATGACACCTGTGTTGTTGTTACG
	Probe	CTGGATCTCCCGTCATCAACGTTGC
	HaALP2-F	ACACTAATGGACCCGGATTCC
	HaALP2-R	GCGATAGTTTGGTTCTGCAGTAAC
	Probe	CCACACGTGAATGACATCCGGCA
	Actin-F	GGCCCCGTCCACAATGA
	Action-R	CCGATCCATACGGAGTACTTCCT
	Probe	ATCAAGATCATCGCGCCCCCAGA
	GAPDH-F	CATTGAAGGTCTGATGACCACTGT
	GAPDH-R	CAGAGGGTCCATCCACTGTCTT
	Probe	CACGCCACCATTGCCACCCA
RNAi	HaCAD-siRNA	GCGUACAAAUAUGGUGAUATT
	HaAPN4-siRNA	GCUGCUGAAAUCGGUCUUATT
	HaALP2-siRNA	GCCGCGGUAACGAUAUCCUTT
	GFP-siRNA	GCGUUGGGAAGUCAAGUUUTT

[a] Restrictions sites used for cloning in pET-30a (+) plasmid are underlined.
